# O062. Post ambulatory surgery headache in patients affected from primary headaches: a comparison with the general population

**DOI:** 10.1186/1129-2377-16-S1-A123

**Published:** 2015-09-28

**Authors:** Giovanni F Manfredi, Francesco De Cesaris, Eugenia Tomas Roldan

**Affiliations:** Centro Day Surgery, Istituto Europeo di Oncologia IRCCS, Milan, Italy; Centro Cefalee, Dip. Scienze della Salute, Università degli Studi di Firenze, Florence, Italy; Unità Ginecologia Preventiva, Istituto Europeo di Oncologia IRCCS, Milan, Italy

## Background

Primary headaches, such as tension-type headache and migraine, are very common. Migraine is one of the most weakening diseases, especially in women[1]. Ambulatory surgery consists in performing some surgical procedures, in selected patients, with discharge from the hospital the same working day[2]. After discharge, some complications may occur at home, above all, pain, nausea, vomiting and headache. We detected the predominance of headache at home in women affected from primary headaches compared to the general population, and its correlation with both anesthesia, the medications usually taken for headache and the drugs given during the intraoperative period.

## Methods

Previously we analyzed data collected within an interval of four months, regarding 1,479 patients (Group A) whom had undergone ambulatory surgery and discharged following the criteria of Post anesthetic discharge score system (PADSS)[3]. At a later stage, we decided to study, in the same way, 64 patients with history of primary headache (Group B - Table 1), treated with a different type of anesthesia (Table 2). Nurses questioned all the patients, during two phone calls at home, both in the evening and in the morning following their discharge from the hospital, concerning the presence of headache and its intensity measured with the Numerical Rating Scale (NRS).

**Table 1:** Table 1

Features	Patients
Female Age 48.2 (±8)	64
***Type of Headache***(IHCD-3)[4]	
Tension-type headache	6
Migraine with aura	5
Migraine without aura	52
Cluster headache	1
***Symptomatic Medications***	
Triptans	15
NSAIDs	23
Acetaminophen	8
Combinations	7
O_2_ therapy	1
None	10
***Preventive Medications***	
β-blockers	8
Amitriptyline	5
Topiramate	3
Vitamins and Supplements	6
None	42

**Table 2:** Table 2

Anesthesia	N.
Local	2
Sedation	2
Local + Sedation (MAC)	6
General	54

We also analyzed data about the personal medications taken for headache, the type of anesthesia, the drugs received during operations for nausea and vomiting such as ondansetron and/or dexamethasone[5] and for postoperative pain, such as acetaminophen, tramadol or ketorolac, individually or combined.

## Results

One hundred and ninety-six patients (13.27%) of Group A and 11 patients (17.19%) of Group B had been suffering from headache at home (Table 3). In Group B, no correlation was shown with usual assumption of headache treatments, the technique of anesthesia, the administration of either prophylaxis for nausea and vomiting (OR: 1.006; 50% CI: 0.52-1.93), or analgesic drugs for the treatment of the postoperative pain (OR: 1.77; 50% CI: 0.54 -7.42). Nevertheless, we noted a higher incidence of headache after the administration of acetaminophen alone (OR: 4.32, 50% IC: 1.36-17.15) but lower incidence with ketorolac both alone or in combination (OR: 0.48, 50% IC: 0.24-1.00), and with dexamethasone (OR: 0.125, 50% IC: 0.02-0.49).

**Table 3:** Table 3

	Group A	Group B
**Patients n.**	1479	64
**Headache n.**	196	11
***P***	*0.132*	*0.171*
***95% IC***	*0.11-0.15*	*0.08-0.28*
*(OR: 1.42, 50% IC: 1.06 - 1.9)*		
**NRS 1-3**	63%	35%
**NRS 4-6**	29%	50%
**NRS 7-10**	8%	15%

## Conclusions

The study showed that headache is a very frequent complication at home, after ambulatory surgery. A higher incidence of headache in the patients already affected from primary headaches was observed. Few correlations, only with some single drug administered during the intraoperative period, were found.

Written informed consent to publication was obtained from the patient(s).
